# Supplementary material for: A gradient of Wnt activity positions the neurosensory domains of the inner ear
Source: eLife. 2021 Mar 11;10:e59540. doi: 10.7554/eLife.59540 (PMC7993990; doi:10.7554/eLife.59540)
Supplement: Transparent reporting form [file elife-59540-transrepform.docx]

***eLife’s* transparent reporting form**

We encourage authors to provide detailed information *within their submission* to facilitate the interpretation and replication of experiments. Authors can upload supporting documentation to indicate the use of appropriate reporting guidelines for health-related research (see [EQUATOR Network](http://www.equator-network.org/%20)), life science research (see the [BioSharing Information Resource](https://biosharing.org/)), or the [ARRIVE guidelines](http://www.plosbiology.org/article/info:doi/10.1371/journal.pbio.1000412) for reporting work involving animal research. Where applicable, authors should refer to any relevant reporting standards documents in this form.

If you have any questions, please consult our Journal Policies and/or contact us: [editorial@elifesciences.org](mailto:editorial@elifesciences.org).

**Sample-size estimation**

- You should state whether an appropriate sample size was computed when the study was being designed
- You should state the statistical method of sample size computation and any required assumptions
- If no explicit power analysis was used, you should describe how you decided what sample (replicate) size (number) to use

Please outline where this information can be found within the submission (e.g., sections or figure legends), or explain why this information doesn’t apply to your submission:

In the presented study we use methods that are qualitative. Any *qualitative* result (for example presence or absence of a sensory organ) is scored in as a number of samples presenting a phenotype out of the number of samples analysed (e.g. x samples out of y tested samples). The severity and variability of the effect determines the number of replicates for the experiment but a minimum of 4 samples for each experimental condition (control, treated) is typically used. Information about the number of samples is indicated in the results section.

**Replicates**

- You should report how often each experiment was performed
- You should include a definition of biological versus technical replication
- The data obtained should be provided and sufficient information should be provided to indicate the number of independent biological and/or technical replicates
- If you encountered any outliers, you should describe how these were handled
- Criteria for exclusion/inclusion of data should be clearly stated
- High-throughput sequence data should be uploaded before submission, with a private link for reviewers provided (these are available from both GEO and ArrayExpress)

Please outline where this information can be found within the submission (e.g., sections or figure legends), or explain why this information doesn’t apply to your submission:

We used at minimum of 4 biological replicates, where the same treatment (concentration of Wnt agonist/antagonist or construct) was applied to different samples. In experiments relying on in ovo electroporation, between 8 to 10 chicken embryos were electroporated with GFP (or other reporter) encoding plasmids and for further analysis only samples with strong fluorescent signal in the inner ear were selected. Information about the number of replicates for each experiment is indicated in the relevant Results section.

**Statistical reporting**

- Statistical analysis methods should be described and justified
- Raw data should be presented in figures whenever informative to do so (typically when N per group is less than 10)
- For each experiment, you should identify the statistical tests used, exact values of N, definitions of center, methods of multiple test correction, and dispersion and precision measures (e.g., mean, median, SD, SEM, confidence intervals; and, for the major substantive results, a measure of effect size (e.g., Pearson's r, Cohen's d)
- Report exact p-values wherever possible alongside the summary statistics and 95% confidence intervals. These should be reported for all key questions and not only when the p-value is less than 0.05.

Please outline where this information can be found within the submission (e.g., sections or figure legends), or explain why this information doesn’t apply to your submission:

Most of our data are qualitative.

The statistical test results and number of samples/cells used for the quantification of Sox2 fluorescence levels in beta-cat GOF transfected cells can be found in the legend of Figure 3 – Suppl. Figure 1.

(For large datasets, or papers with a very large number of statistical tests, you may upload a single table file with tests, Ns, etc., with reference to sections in the manuscript.)

**Group allocation**

- Indicate how samples were allocated into experimental groups (in the case of clinical studies, please specify allocation to treatment method); if randomization was used, please also state if restricted randomization was applied
- Indicate if masking was used during group allocation, data collection and/or data analysis

Please outline where this information can be found within the submission (e.g., sections or figure legends), or explain why this information doesn’t apply to your submission:

The embryos were allocated randomly to control and experimental groups for in ovo electroporation and treatments of organotypic cultures with pharmacological agents. The allocation of embryos to different groups and the analysis of the results were not performed blindly.

**Additional data files (“source data”)**

- We encourage you to upload relevant additional data files, such as numerical data that are represented as a graph in a figure, or as a summary table
- Where provided, these should be in the most useful format, and they can be uploaded as “Source data” files linked to a main figure or table
- Include model definition files including the full list of parameters used
- Include code used for data analysis (e.g., R, MatLab)
- Avoid stating that data files are “available upon request”

Please indicate the figures or tables for which source data files have been provided:
